# Comparison of affective temperament, parental bonding, and intelligence between individuals who chose medical and non-medical professions

**DOI:** 10.3389/fmed.2025.1647460

**Published:** 2025-09-18

**Authors:** Hirofumi Hirakawa, Takeshi Terao, Kentaro Kohno, Akari Sakai, Nobuko Kawano

**Affiliations:** ^1^Department of Neuropsychiatry, Faculty of Medicine, Oita University, Yufu, Japan; ^2^Oita Occupational Health Management Center, Nishinihon Occupational Health Center, Oita, Japan; ^3^Department of Psychology, Faculty of Welfare and Health Sciences, Oita University, Oita, Japan

**Keywords:** professional choice, medical doctors, nurses, psychologists, paternal care

## Abstract

**Background:**

Professional choice is an important aspect of one’s life and is associated with biopsychosocial and economic factors. Medical and co-medical professional choices may involve a noble intention to contribute to patients. Thus, this study aimed to investigate the association between medical and co-medical professional choices and affective temperament, parental bonding, and intelligence.

**Methods:**

The dataset included 130 individuals (19 with medical or co-medical professional choices and 111 without). Data on participants’ demographics, intelligence levels, affective temperament, and parental bonding were collected and subsequently compared among the two groups using an unpaired t-test and *χ*^2^ test. Thereafter, binomial logistic regression analysis using the likelihood ratio and forward method was performed, with medical or co-medical professional choice as the dependent variable and potentially significant variables (*p* < 0.2) in the above *t*-test or *χ*^2^ test as independent variables.

**Results:**

Only higher paternal care was significantly associated with medical and co-medical professional choices.

**Conclusion:**

Our study findings suggest that paternal care is associated with medical or co-medical professional choices. Further prospective studies are required to determine causal relationships and investigate other factors related to such choices, given the non-association of all other variables in the study.

## 1 Introduction

The notion that temperament plays a role in professional choice goes back to Aristotle, who wondered why poets, philosophers, and politicians all had a melancholic temperament (1). In 1931, Kretschmer wrote a fascinating monograph on individuals of genius, largely German poets and musicians, and based on the existing biographical data, declared them cyclothymic (1). Since World War II, the most impressive findings have been the association of cyclothymic temperament with artistic creativity and that of hyperthymic temperament with leadership (2). With respect to creativity, students enrolled in arts schools (i.e., those preparing for creative artistic professions) exhibited higher scores on cyclothymic, hyperthymic, and irritable temperaments compared with university students in disciplines that are expected to lead to professions primarily requiring the application of the learned rules (e.g., law, engineering, and foreign language studies) (3). Moreover, tango dancers, representing one of the most characteristic Argentine folk dance–musical repertoires, showed higher scores on hyperthymic and irritable temperaments compared with the general population (4). Although the relationship between temperament and career choice has been noted since ancient times, modern research has revealed that this relationship has been studied more concretely, particularly among health professionals.

Professional choice is an important aspect of one’s life and is associated with both biopsychosocial and economic factors. Especially, medical or co-medical professionals may have noble intentions to contribute to patients. Akiskal et al. (1) showed that physicians had nearly twice as much depressive temperament as controls (21% vs. 12%) and nearly twice as many obsessive-compulsive traits (32% vs. 17%). Figueira et al. (5) showed that psychology and nursing students predominantly presented with depressive and anxious temperaments, whereas medical students had the lowest scores of hyperthymic and cyclothymic temperaments. Jaracz et al. also confirmed that nurses had a higher anxious temperament than firefighters, athletes, paramedics, and civil servants (6, 7). On the other hand, emergency medicine professionals exhibited elevated levels of hyperthymic temperament compared with other temperaments. This finding suggests that hyperthymic temperament may represent a desirable trait in emergency medicine professionals who must work under high levels of risk and perform complex medical tasks under extreme conditions (8).

However, to the best of our knowledge, no study has investigated the association between parental bonding and medical or co-medical professional choices. It seems plausible that higher levels of maternal and paternal care can foster the noble intention to contribute to patients with the aspiration to be a medical or co-medical professional. Finally, a certain level of intelligence is also required to achieve medical or co-medical education. Therefore, we hypothesized that medical or co-medical professionals would have higher depressive and anxious temperaments, higher maternal and paternal bonding, and higher intelligence than the others. This study aimed to test this hypothesis.

## 2 Subjects and methods

### 2.1 Subjects

We used data from a previous study (9) on psychotherapy in apparently healthy participants. In the previous study, the inclusion criterion was individuals aged ≥ 20 years who provided written informed consent. Participants were recruited via electronic bulletin boards, physical bulletin boards, and flyers. Individuals with serious psychiatric disorders, as determined by the mini-international neuropsychiatric interview (M.I.N.I.), were excluded. The Institutional Review Board of Oita University Faculty of Medicine approved the trial (number B16-023). All participants provided written informed consent. Although the previous study (9) had 137 subjects, our previous opt-out study (10) excluded seven subjects and created the dataset of 130 subjects because of missing data. In this opt-out study, we used the dataset of 130 subjects, which included 108 women and 22 men, with a mean age of 49.3 years (SD = 12.1). This opt-out study was approved by the ethics committee of the Oita University Faculty of Medicine on 12 June 2023 (approval number 2536).

### 2.2 Medical professionals

We defined medical or co-medical staff as doctors, nurses, public health nurses, psychotherapists, occupational therapists, social workers, and medical students, as they work for patients and families; medical students were included as they aspire to become doctors. We called them the medical group and the others the non-medical group.

### 2.3 Measures

#### 2.3.1 Affective temperament

We used the Temperament Evaluation of Memphis, Pisa, Paris, and San Diego-auto questionnaire (TEMPS-A) to assess participants’ affective temperament. It is a 110-item true–false questionnaire that assesses five temperament dimensions: depressive, cyclothymic, hyperthymic, irritable, and anxious (11). TEMPS-A has been translated into Japanese, and its reliability and validity have been sufficiently established (12).

#### 2.3.2 Parental bonding style

The Parental Bonding Instrument (PBI) is a retrospective self-assessment measure of parental rearing attitudes experienced in childhood (13). The PBI consists of 25 items divided over two subscales of “care (12 items)” and “overprotection (13 items).” A high score for “care” indicates more appropriate rearing (less rejection and indifference), whereas a high score for “overprotection” indicates inappropriate overprotection (less encouragement of self-reliance). Each item is assessed on a four-point Likert scale. The PBI has been translated into Japanese with well-established reliability and validity (14, 15).

#### 2.3.3 Intelligence

The National Adult Reading Test (NART) (16) is a widely used measure of premorbid IQ in English-speaking patients with dementia. The Japanese version of the NART (JART) uses 50 irregular Japanese words, all of which are kanji (ideographic script) compound words, which have a well-established reliability and validity (17). The JART has also been used in studies assessing IQ (particularly Verbal IQ) in healthy adults without a medical history of psychiatric disorders (18, 19).

### 2.4 Data analysis

First, demographic data, affective temperament, parental bonding, and intelligence scores were compared among the two groups (medical and non-medical groups) using an unpaired *t*-test or *χ*^2^ test. Second, binomial logistic regression analysis using the likelihood ratio and forward method was performed, with medical professional choice as the dependent variable and potentially significant variables (*p* < 0.2) in the above *t*-test or *χ*^2^ test as independent variables. We used IBM SPSS Statistics Ver 27.

## 3 Results

Among the 130 participants, 19 were from the medical group, including seven nurses, three social workers, two psychotherapists, two occupational therapists, two public health nurses, two medical students, and one medical doctor. The remaining 111 individuals were from the non-medical group, including 27 office workers, 11 housewives, 10 part-time workers, 8 public servants, etc. The medical group included 18 women and only 1 man, whereas the non-medical group included 90 women and 21 men. However, gender was not significantly different (χ^2^ = 2.15, *p* = 0.142). The medical group also had significantly higher paternal care scores than the non-medical group (Table 1).

**Table 1:** Comparison of age, gender, affective temperament, parental bonding, and intelligence between medical and non-medical groups.

Variables	Medical group (*n* = 19)	Non-medical group (*n* = 111)	
Age, mean (SD, *n*)	44.53 (11.38, 19)	50.05 (12.1, 111)	0.06
Gender (male, female)	1, 18	21, 90	0.12
TEMPS-A mean (SD, *n*)			
Depressive temperament	7.33 (3.02, 18)	8.77 (3.56, 111)	0.1
Cyclothymic temperament	4.61 (3.72, 18)	4.9 (4.3, 110)	0.78
Hyperthymic temperament	5.67 (4.53, 18)	5.01 (3.16)	0.44
Irritable temperament	2.5 (3.18, 18)	3.02 (3.17, 110)	0.52
Anxious temperament	5.65 (4.92, 17)	6.91 (5.37,111)	0.36
PBI mean (SD, *n*)			
Paternal care	25.72 (8.05, 18)	20.86 (8.79, 106)	0.03
Paternal overprotection	10.56 (6.23, 18)	11.48 (7.0, 106)	0.6
Maternal care	26.11 (4.87, 18)	23.95 (7.88, 106)	0.12
Maternal overprotection	10.56 (5.07, 18)	11.65 (7.77, 109)	0.44
JARTIQ mean (SD, *n*)			
Intelligence	108.79 (6.34, 18)	109.78 (7.78, 111)	0.59

JART, Japanese version of the national adult reading test; PBI, Parental Bonding Instrument; s.d., Standard deviation; TEMPS-A, Temperament Evaluation of Memphis, Pisa, Paris, and San Diego-auto questionnaire.

As previously mentioned, binomial logistic regression analysis using the likelihood ratio and forward method was performed, with medical professional choice as the dependent variable and potentially significant variables, which showed *p* < 0.2 in the *t*-test or *χ*^2^ test as independent variables they were gender, age, depressive temperament, paternal care, and maternal care. As a result, only paternal care remained significant in the final model (Table 2).

**Table 2:** Binomial logistic regression analysis for medical professional choice as dependent variable.

Variables	*β*	SE	Odds ratio	95% CI	*p* value
Paternal care	0.07	0.036	1.073	1.00–1.15	0.048

SE, Standard error; CI, Confidence interval. The significance of the model, *p* = 0.035.

## 4 Discussion

This study aimed to investigate whether medical or co-medical professional choices are associated with individuals’ affective temperament, parental bonding, and intelligence. Only paternal care was significantly associated with medical and co-medical professional choice, and this did not change after binomial logistic regression analysis.

Based on the previous studies (1, 5–7), we hypothesized that medical or co-medical professionals may have higher depressive and anxious temperaments, higher maternal and paternal bonding, and higher intelligence than the others, but this was mostly rejected. However, our hypothesis was only partially supported by the association between paternal bonding and medical or co-medical professional choice. To the best of our knowledge, this is the first study to demonstrate such an association. Interestingly, another study showed that maternal but not paternal care was significantly associated with educational level and marital status (20). It is uncertain why paternal care was significantly associated with medical or co-medical professional choice. Although highly speculative, paternal care may foster the noble intention of physically and/or psychologically contributing to weak individuals, such as patients. However, the reason for the non-association between medical or co-medical professional choice and depressive or anxious temperaments or intelligence may possibly be the heterogeneity of medical professionals in this study.

This study has some limitations that must be considered. First, this was a cross-sectional, retrospective study, and causal relationships could not be determined. Second, the sample size was relatively small, which precluded to establish a definite conclusion. Third, the JARTIQ does not measure total intelligence. Finally, this study focused only on psychological aspects. Biological, social, economic, and cultural aspects should also be considered while studying these associations. In particular, parental occupation may also have influenced individuals’ professional choices (e.g., parents’ occupations may reflect their financial situation, and children may become familiar with or aspire to their parents’ professions). However, this study did not take parental occupation into account.

## 5 Conclusion

The present findings suggest that paternal care may be associated with medical or co-medical professional choices. Further prospective studies are required to determine causal relationships and investigate other factors related to such choices, given the non-association of all other variables in the study.

## Data Availability

The data analyzed in this study is subject to the following licenses/restrictions: because we are making other papers using the data. Requests to access these datasets should be directed to TT, terao@oita-u.ac.jp.
